# Intraseason Changes in Vertical Jumps of Male Professional Basketball Players

**DOI:** 10.3390/ijerph20065030

**Published:** 2023-03-13

**Authors:** Álvaro de Pedro-Múñez, Tania Álvarez-Yates, Virginia Serrano-Gómez, Oscar García-García

**Affiliations:** Laboratory of Sport Performance, Physical Condition and Wellness, Faculty of Education and Sport Sciences, University of Vigo, Campus A Xunqueira s/n, 36005 Pontevedra, Spain

**Keywords:** countermovement jump, squat jump, arm swing countermovement jump, neuromuscular performance, power, lower limbs

## Abstract

This study aimed to analyze basketball players’ jumping bhavior in the Squat Jump (SJ), Countermovement Jump (CMJ), and Free Arm Swing CMJ (CMJ Free) during a professional basketball season and check if it is modulated by the players’ specific playing position, the time played on court, and the different leagues. Fifty-three male professional basketball players were assessed in three different moments of the season through SJ, CMJ, and CMJ Free. Between the beginning of pre-season (1st assessment) and the second round of the season (3rd assessment), there was a strong increase in performance in the three jumps (SJ Height: 5.6%, η^2^_P_ = 0.234, *p* = 0.007; CMJ Height: 5.1%, η^2^_P_ = 0.177, *p* = 0.007; CMJ Free height: 4.11%, η^2^_P_ = 0.142, *p* = 0.01). There was also a significantly large increase in SJ and CMJ between the 2nd and 3rd assessments and in the CMJ Free between the 1st and 2nd assessments. No significant interactions were found between jumping performance and the group factors (players’ specific playing position, time played on court, and league). In conclusion, SJ, CMJ, and CMJ Free performance strongly increases between 1st and 3rd assessment, without being influenced by the specific playing position or the minutes played per game.

## 1. Introduction

There are many important characteristics that determine performance in basketball, such as jumping, throwing, and anaerobic endurance capacities [1]. However, a player’s jumping capacity seems crucial since more than 50 explosive jumping actions can be performed per basketball game [2], which is one every fifty-two seconds in professional basketball players [3]. These jumping actions have been estimated at around 1.5% of the total time played [4]. Therefore, basketball players’ neuromuscular properties are of great importance due to their key role in the jumping capacity.

In fact, this jumping capacity is highly used in draft combine tests for the identification and selection of players for national basketball teams. Arede et al. [5] highlighted that the Abalakov Jump Peak Power, together with the predicted adult height of under 14 basketball players, can successfully discriminate selected from non-selected players for the Portuguese basketball team. García-Rubio et al. [6] pointed out the predictive ability of the standard vertical jump and running vertical jump for NBA draft position and game performance. In this line, Cui et al. [7] found that drafted NBA players outperformed the undrafted ones in vertical jump height and reach. It should be noted that greater jumping capacity has been reported in more competitive leagues, indicating that there is probably an advantage for the best jumpers to compete in higher leagues [8]. Hence, the different match-play demands of basketball in different levels of competition or different leagues may be reflected in the players [9].

Basketball players show differences depending on their specific playing position, both in physical and game actions. Height, wingspan, and leg power can serve as key determinants for being drafted as guards, while agility and speed is important for power forwards and centers [7]. However, in terms of game actions, such as the number of jumps performed per game, Abdelkrim et al. [10] reported 41 jumps for point guards and guards and 49 jumps for pivots, while Ostojic et al. [11] reported 59.7 ± 9.6 for guards, 57.8 ± 6.7 for wings, and 54,6 ± 11 for pivots. These jumping variables have been demonstrated to be extremely important for basketball competition performance, as strong correlations have been found between countermovement jump (CMJ) performance and time on the court [12]. In addition, jumping performance appears to be related to performance level since significant correlations have been obtained between a player’s division and jump height [13].

Changes in jumping performance during a basketball season have been previously reveled by different authors. Aoki et al. [14] reported increases in CMJ (8.8% ± 6.1, ES > 0.6) and Squat Jump (SJ) (14.8% ± 10.2, ES > 0.8) between pre-season and in-season. Gonzalo-Skok et al. [15] concluded that 8 weeks of eccentric overload training induced substantial improvements in all functional performance tests (COD tests, a 25-m linear sprint test, unilateral multidirectional jumping tests). In addition, playing time appears to enhance jumping capacity [16].

However, it seems interesting to carry out an analysis on how basketball players’ jumping capacities, in terms of height and flight time, change during a professional basketball season, since, to our knowledge, there are no longitudinal studies that analyze a large period of a competition season. Hence, we hypothesize that the jumping capacity of professional basketball players could improve throughout the season without depending on the specific basketball-playing position of the player, the time played on court, and the players’ leagues. Therefore, the aim of this study was to analyze the behavior of basketball players’ jumping capacities in the squat jump (SJ), countermovement jump (CMJ), and free arm swing CMJ (CMJ Free) during a professional basketball season and to check if it is modulated by the specific basketball-playing position, the time played on court, and the players’ leagues.

## 2. Materials and Methods

### 2.1. Study Design

A longitudinal design was used to determine basketball players’ neuromuscular behaviors throughout a professional basketball season. Basketball players were assessed in three different moments within the season: (1) 1st assessment during the first week of the pre-season, (2) 2nd assessment during the first month of the competitive season (in October), and (3) 3rd assessment during the second month of the second round (in February).

Jumping testing was carried out on court, after a protocolized 12 min warm-up. This warm-up included shooting, multi-direction movements, dynamic soft stretching, core and glute activation, ballistic stretching, agility, and speed. Then, plyometric stimulus was introduced by performing an SJ, a CMJ, and a CMJ Free. Players performed two attempts for each jump, separated by a self-preferred rest period. The testing protocol was performed in the following order: SJ, CMJ, and FJ. Players were encouraged in each jump to reach the highest possible height. The highest jumping value was used for further analyses. To determine measurement reliability, 11 players were randomly selected to carry out a second measurement. These data were used to calculate the intraclass correlation coefficient (ICC) and coefficient of variation (CV).

### 2.2. Participants

Fifty-three male professional basketball players from the German Basketball Bundesliga (BBL) and Spanish Basketball League (LEB) were evaluated during a season (age: 24.5 ± 3.4 years; body mass: 94.9 ± 10.8 kg; height: 197 ± 9.5 cm; fat%: 8.9 ± 1.8). Sample size was calculated using G*power v3.1.9.4 for Windows (Heinrich-Heine-Universität Düsseldorf, Germany) for ANOVA repeated measures (3 assessment), using factor groups based on an effect size of 0.25, a power of 0.95, and an alpha level of 0.05. This power analysis determined that the needed sample size should be 44 players.

The 53 players were divided according to their specific playing position: 33 guards (point guards, shooting guards, and small forwards) and 20 centers (centers and power forwards), according to the minutes played on court per game during the season (more than 20 min per game or less than 20 min), and according to their provenance league (31 players in the BBL and 22 players in the LEB). No player had physical limitations, health problems, or musculoskeletal injuries that could compromise the testing protocol. Players who reported an injury during data collection were excluded from the study. All participants were informed regarding the nature, aims, and risks associated with the testing protocol. A signed informed consent was obtained from all basketball players. This study was approved by the local research ethics committee and conducted in accordance with the Declaration of Helsinki (64th WMA General Assembly, Fortaleza, Brazil, 2013).

### 2.3. Procedure

Jumping capacity was inferred by flight time (FT) using a Chronojump BoscoSystem contact platform (v.1.7.0 for Windows, CHRONOJUMP Boscosystem^®^, Barcelona, Spain). This tool has been previously validated, demonstrating good reliability for measuring vertical jumps (ICC = 0.95) [17]. Players were instructed to land as normal, that is, with an ankle extension to reduce the impact forces on the joints, as they would during any jump executed during trainings and/or games. If any player landed with the ankles, knees, or hips bended, the jump would be considered invalid, and they would have to perform a new jump. If any doubt arose regarding jumping technique, it would be also a reason to repeat the jump.

The squat jump (SJ) was performed following Toumi’s et al. [18] protocol. The players were asked to go down to a semi squat position, maintaining this position for 2 s until the evaluator said “GO!”. Each player was allowed to self-select their knee angle, since knee flexion differences between 90°, 100°, and the self-selected are trivial [19]. Nevertheless, almost every player chose a depth within this range. No pre-stretching or countermovement was allowed before the concentric phase, and during the jump, the arms had to be maintained on the hips to avoid taking advantage of upper limbs. If any of these circumstances were perceived, the jump had to be preformed again. In this way, we eliminated the elastic properties, isolating the concentric strength.

The countermovement jump (CMJ) was carried out similarly to the SJ, without maintaining knee flexion position and performing a fast and explosive countermovement [18,19,20]. Players maintained their hands on their hips, went down at a preferred place, and then tried to jump as high as possible. With this jump we tested not only the concentric strength, but also the elastic properties of the muscle–tendon structure of the player.

Free arm swing CMJ (CMJ Free) was performed with the only rule being that the player must reach the maximum possible height [21]. To achieve it, they could use their upper limbs, reach their prefer depth in the impulse phase, and perform this phase faster or slower. We tried to analyze not only the concentric strength and the elastic properties, but also the coordination specific skills.

### 2.4. Statistical Analysis

The Kolmogorov–Smirnov test indicated that the data was normal, linear, and homoscedastic. The measurements’ relative reliabilities were analyzed using the intraclass correlation coefficient (ICC) by single measures, the 2-way mixed effects model, and absolute agreement [22]. Coefficient of variation (CV), via Hopkins’ log-transformed data, was used for absolute reliability analysis. A mixed ANOVA using repeated measures was used to detect changes in a basketball player’s jumping performance during the season. The 3 assessment moments (assessment 1, 2 and 3) were used as repeated measure variables, while the players’ specific playing positions (guards or centers), the time played on court per game (>20 min or <20 min), and the leagues (BBL or LEB) were used as the group factors (*i.e.*, *3 assessments × 2 specific playing positions × 2 played times × 2 leagues*). Bonferroni post hoc tests with adjustment for 95% confidence intervals were used to compare the main effects and identify significant individual differences. The effect sizes in repeated measures of ANOVA were reported as partial eta square (η^2^_p_) and interpreted as small (0.01), moderate (0.06), or large (0.14) [23].

## 3. Results

All jump measurements have shown a good ICC relative reliability (range 0.91 to 0.94; 95% confidence interval = 0.76–0.99). Moreover, the minimum CV was 3.8% for CMJ, while the maximum for SJ was 5.5%, indicating a very good absolute reliability for all jump measurements.

As can be seen in Table 1, SJ showed significant increase in performance between the 1st and 3rd assessments, with a large effect size (SJ Height: 5.6%; η^2^_P_ = 0.234; *p* = 0.008), as well as between the 2nd and 3rd assessments (SJ Height: 4.0%; η^2^_P_ = 0.234; *p* = 0.01). However, these changes were not significant between the 1st and 2nd assessments (*p* = 0.458). The interactions *SJ × specific playing position* (F = 2.027; *p* = 0.144; η^2^_P_ = 0.088), *SJ × min/game* (F = 0.633; *p* = 0.536; η^2^_P_ = 0.029), and *SJ × league* (F = 0.844; *p* = 0.437; η^2^_P_ = 0.039) did not show significant differences, nor did any other interactions between *SJ × specific playing positions × min/game × league*. Therefore, changes in SJ performance during the season do not seem to be influenced by the player’s specific playing position, the minutes played, or by the different leagues.

Similarly, there was a large increase in CMJ performance between the 1st and 3rd assessments (CMJ Height: 5.1%; η^2^_P_ = 0.177; *p* = 0.007) and between the 2nd and 3rd assessments (CMJ Height: 3.8%; η^2^_P_ = 0.177; *p* = 0.01). As in the SJ, these changes were also not significant between the 1st and 2nd assessments (*p* = 0.451). The interactions *CMJ × specific playing position* (F = 0.012; *p* = 0.988; η^2^_P_ = 0.000), *CMJ × min/game* (F = 1.448; *p* = 0.245; η^2^_P_ = 0.055), and *SJ × league* (F = 0.264; *p* = 0.769; η^2^_P_ = 0.001) did not show any significant differences either, nor did any other interactions between *CMJ × specific playing positions × min/game × league*. Hence, changes in CMJ performance during the season do not seem to be influenced by the player’s specific playing position, the minutes played, or by the different leagues (see Table 1).

In addition, players also increased their performance significantly in free arm swing CMJ between the 1st and 2nd assessments, with a large effect size (CMJ Free Height: 4.47%; *p* = 0.01; η^2^_P_ = 0.142), and the 1st and 3rd assessments (CMJ Free Height: 4.11%; *p* = 0.01; η^2^_P_ = 0.142;). However, following the trend of the abovementioned jumps, no significant changes were observed between the 2nd and 3rd assessments (*p* = 0.799) (see Table 1). Neither has been found among the interactions *CMJ Free × specific position* (F = 1.089; *p* = 0.342; η^2^_P_ = 0.046), *CMJ Free × min/game* (F = 0.153; *p* = 0.859; η^2^_P_ = 0.007), *CMJ Free × league* (F = 0.372; *p* = 0.691; η^2^_P_ = 0.016), nor in the set of interactions (*CMJ Free × specific playing positions × min/game × league)*. Therefore, changes in free arm swing CMJ performance during the season do not seem to be influenced by the player’s specific playing position, the minutes played, or by the different leagues (see Table 1).

Regarding the minutes played on the court per game during the season, 51.1% of our basketball players played more than 20 min per game, while the 48.9% played less than 20 min. No significant differences were found, either in SJ (F = 0.069; *p* = 0.809; η^2^_P_ = 0.003), CMJ (F = 0.137; *p* = 0.714; η^2^_P_ = 0.005), or CMJ Free (F = 0.723; *p* = 0.440; η^2^_P_ = 0.030), between players who played <20 min or >20 min per game during the season (see Table 2). 

Concerning to the basketball leagues, players from the BBL and LEB showed a similar behavior in jump performance during the season. Although BBL players showed greater performance in all jumps throughout the competition season, (SJ: 1st = 36.86 vs. 40.87; 2nd = 38.32 vs. 40.54; 3rd = 39.59 vs. 42.60 cm; CMJ: 1st = 41.17 vs. 43.13; 2nd = 41.93 vs. 43.45; 3rd = 43.07 vs. 45.45 cm; CMJ Free: 1st = 47.83 vs. 52.03; 2nd = 50.50 vs. 54.05; 3rd = 50.43 vs. 53.81 cm), no significant differences were found between both leagues.

## 4. Discussion

Our main findings indicate that between the beginning of the pre-season (1st assessment) and the second round, and, specifically, the second month of the regular professional basketball season (3rd assessment), there was a strong increase in the players’ SJ, CMJ and free arm swing CMJ performances. In addition, there was a large increase in SJ and CMJ between the first month of the competitive season (2nd assessment) and the second month of the second round of the basketball season (3rd assessment). Finally, there was also a large increase in CMJ free height between the beginning of the pre-season (1st assessment) and the first month of the competitive season (2nd assessment). We have hypothesized that the jumping capacity of professional basketball players could improve throughout the season, so, in view of our findings, our hypothesis seems to be confirmed. However, these changes are not influenced by the players’ specific playing positions, the average minutes played per game, or the different leagues.

A few decades ago, the belief was that team sport players could only hope to maintain their fitness level during season. In 1991, Hoffman et al. [24] analyzed the physical performance changes during the season, showing that, by midseason, squat strength, vertical jump, and sprint performance were significantly lower, with a trend of decreased physical performance to the end of the season. For this reason, maintaining physical performance in team sports during the season was a challenge, especially regarding neuromuscular variables. Currently, we can observe how specific training and playing time can contribute to the maintenance of performance levels during the season. Complex training, where different contents are mixed to improve maximum strength, power zone, speed, and over speed (e.g., assisted jumps) seem to improve neuromuscular performance [25,26]. This could be one of the keys to the improvements shown in our professional basketball players. These improvements during the season may seem moderate, but it is worth noting that the higher the player’s baseline level, the more difficult it was to improve neuromuscular performance [27,28]. This fact can be seen in how athletes with less training in their backgrounds tend to show greater improvements in response to training interventions.

Specifically, our results have shown that basketball players experienced significant improvements during the season in SJ. This could be because the concentric strength of basketball players, which is the main factor influencing SJ’s performance, also increases during the competitive season. Like us, González et al. [15] also found improvements in SJ power during the NBA competitive season. Hence, it seems that the most specific movements can be improved during the competitive season with adequate strength training programs [29]. That is, a sufficient volume of heavy resistance training should be applied during the microcycles, although some external factors could challenge these interventions (e.g., training and competition calendars, hits suffered during games or trainings, body discomfort, etc.).

As for the CMJ performance, it also improves significantly throughout a professional basketball player’s season. This fact has already been observed in NBA starters, who are more likely to maintain performance over the season [15]. However, these latter authors only focused on power variables, instead of jumping time and height like us, hence, these data should be compared with caution. It seems that muscle–tendon elastic properties are not only maintained but improved during the season. Although, basketball players’ jumping capacities seem to be influenced by their strength training programs, since several authors have pointed that muscle–tendon stiffness is increased by strength training [29], which is usually composed of heavy traditional resistance exercises that produce more significant changes in advanced individuals [28]. Gonzalo-Skok et al. [14] found, in amateur/semiprofessional team-sport players, that an 8-week eccentric overload strength training during the season produced greater improvements in CMJ when performing a training program based only on bilateral vertical exercises (6,6%) rather than including unilateral multi-directional exercises (5,8%). These improvements are smaller than those reported by Aoki et al. [13], even though they are greater than ours. Yet, we need to be aware of the players’ different levels of performance.

Likewise, basketball players’ performances in CMJ Free also increased significantly through the season, with a large effect size, especially between pre-season (1st assessment) and the beginning of the first round of the season (2nd assessment). However, CMJ Free performance stabilizes in the second round (3rd assessment). Hence, it seems that a specific jump, as the CMJ Free that involves concentric strength, elastic properties, specific angular speed, ROM, and technique, is the one that most benefited from the training process. Aoki et al. [9] also found, as us, an increase in professional basketball players’ jumping capacities after the pre-season. However, they only analyzed SJ and CMJ, showing, like us, improvement in both jumps, yet with a moderate effect size (CMJ: 8.8% ± 6.1, ES > 0.6; SJ: 14.8% ± 10.2, ES > 0.8). These differences could be due to the difference level of performance at baseline between our players and Aoki et al.’s [13] (SJ: 34.9 vs. 38.44cm; CMJ: 38,1 vs. 42,09 cm), since highly trained athletes with long experience in strength training find it more difficult to keep improving, compared to less trained athletes [27,28]. Nonetheless, it should be noted that, during the off-season, the volume of 5 × 5 training is very low, but then, as the season progresses, it increases considerably. We find it reasonable to think that the number of free arm swing CMJs performed during pre-season and in-season was much higher. Therefore, following these data, the volume difference between the SJ, CMJ, and CMJ Free was similar in the off-season, but, in the pre-season and in the season, the CMJ Free received much more training volume than the SJ and CMJ. In addition, it has been suggested that the aerobic fitness and the ability to sustain high intensity efforts in Italian basketball players from Division I and II are improved during the preparation period, but the same does not occur with vertical jump variables [30]. Hence, it seems that the neuromuscular properties are more difficult to develop than the metabolic ones, especially in professional players. Nevertheless, these latter authors did find increases in force and power output during vertical jumps across the season, yet these were slight-to-moderate (ES: 0.20–0.73).

It is also important to note that players’ mood states could possibly have also influenced jumping performance during the season, especially with regard to their efforts to perform the jumping tests. This fact has already been pointed out by Hoffman et al. [31], who also concluded that mood state vigor may be reflective of team performance. In addition, it seems that when athletes feel stressed, their locus of control shifts to external and they feel little control over their circumstances, which can lead to reduced motivation [32]. This could have reduced the magnitude of the neuromuscular improvements that the players showed in the consecutive test, due to a lack of maximum effort.

However, neither players’ specific playing positions (guards vs. centers) nor the minutes total played on the court per game (>20 min vs. <20 min) seemed to modulate professional basketball players’ jumping performance changes during the season. This has been previously highlighted by Altavilla et al. [33] and Pehar et al. [34]. However, some differences have been found between playing position and the number of vertical jumps performed between guards and pivots (41 vs. 44 jumps/game, respectively) [35]. In addition, running jumps appear to show a high capacity to discriminate between positions [34]. In this line, is has been pointed out that guards can be differentiated from forward/centers by their strength and power characteristics [30]. Nevertheless, in NBA starter players, who have shown a higher average of minutes player per game than non-starters (27.8 ± 6.9 vs. 11.3 ± 7.0 min, respectively) showed greater improvements over the course of the season [15]. These differences may be due to the significant differences in playing time between NBA starters and non-starters, while, in our sample, playing time was distributed much more evenly.

The analysis between different country leagues showed that BBL players have a higher jumping performance than LEB players in the three jumps (SJ, CMJ, and CMJ Free). This may be due to the higher performance level of the German league players (at least theoretically) since players from more competitive leagues show greater jumping performance [8]. However, this statement should be analyzed in depth in future studies.

The main limitation of this study is that three assessments could not be enough to analyze all the changes can occur during a professional basketball season. In addition, although we analyzed vertical jumps with different key performance factors, we could have added other types of tests, such as jumps with a horizontal component (e.g., running test, due to its high ability to discriminate between positions [34]). We strongly believe that more longitudinal studies in professional basketball are needed, with a greater number of assessments, a wider testing battery composed of different natures of tests (e.g., change of direction, speed, agility, etc.), which could also help to analyze players´ changes in force velocity profiles. In addition, an exhaustive control of training and competition load could be adequate to better explain the changes that occur in professional basketball jumping performance. Another interesting variable to explore would be the anthropometric characteristics of the players and their relationship with jumping performance, in line with Altavilla et al. [33], or analyzing through running max vertical jumps, in line with García-Rubio et al. [6] and Pehar et al. [34]. Therefore, we recommend that strength and conditioning coaches analyze the number of jumps performed by their players during the different stages the season (off-season, pre-season, and in-season) to build a jump performance baseline and to better understand the longitudinal changes in the SJ, CMJ and CMJ Free capacity. These findings can be useful for basketball coaches and physical trainers, as the use of CMJ Free as a regular test can help to determine jumping performance improvements throughout professional basketball players’ seasons.

## 5. Conclusions

Between the beginning of the pre-season and the second round a regular professional basketball season, there was a strong increase in players’ SJ, CMJ and free arm swing CMJ performances, without being influenced by the specific position of the player or the minutes played per game. German and Spanish basketball leagues show a similar trend in jumping performance behavior during the season. Basketball coaches and strength and conditioning coaches could use these jumps to assess jumping performance throughout the season and establish a performance profile based on this parameter.

## Figures and Tables

**Table 1:** Basketball players’ jumping assessments during the season based on playing position (Mean ± SD).

Jumping Parameter	Position	1st Assessment	2nd Assessment	3rd Assessment
SJ Height	Guards	39.91 ± 5.42	41.08 ± 4.65	41.73 ± 4.66
	Centers	36.69 ± 4.30	36.20 ± 4.68	39.60 ± 4.41
	Overall	38.80 ± 5.22	39.39 ± 5.15	40.99 ± 4.61 *^++^
SJ FT	Guards	0.573 ± 0.037	0.574 ± 0.032	0.583 ± 0.029
	Centers	0.548 ± 0.035	0.544 ± 0.040	0.568 ± 0.030
	Overall	0.558 ± 0.034	0.564 ± 0.033	0.578 ± 0.031 *^++^
CMJ Height	Guards	42.92 ± 4.61	43.61 ± 4.91	45.33 ± 5.11
	Centers	41.21 ± 5.34	41.40 ± 5.50	42.90 ± 3.82
	Overall	42.30 ± 4.88	42.81 ± 5.16	44.44 ± 4.77 *^++^
CMJ FT	Guards	0.591 ± 0.031	0.596 ± 0.033	0.607 ± 0.030
	Centers	0.579 ± 0.043	0.580 ± 0.043	0.591 ± 0.031
	Overall	0.586 ± 0.034	0.590 ± 0.036	0.601 ± 0.030 *^++^
CMJ Free Height	Guards	50.09 ± 5.74	52.78 ± 5.96	52.83 ± 6.19
	Centers	51.32 ± 5.96	52.70 ± 8.04	52.09 ± 5.04
	Overall	50.50 ± 5.75	52.76 ± 6.60	52.58 ± 5.76 ^+^*
CMJ Free FT	Guards	0.638 ± 0.034	0.657 ± 0.033	0.658 ± 0.029
	Centers	0.646 ± 0.041	0.656 ± 0.057 ^+^	0.652 ± 0.044
	Overall	0.639 ± 0.035	0.657 ± 0.041 ^+^	0.656 ± 0.033 ^+^*

FT: flight time; CMJ: countermovement jump; + *p* ≤ 0.01 between 1st and 2nd assessment; * *p* ≤ 0.01 between 1st and 3rd assessment; ++ *p* ≤ 0.01 between 2nd and 3rd assessment.

**Table 2:** Basketball players’ jumping assessments during the season based on the time played on the court (Mean ± SD).

Jumping Parameter	Time Played	1st Assessment	2nd Assessment	3rd Assessment
SJ Height	+20 min	38.14 ± 6.18	38.56 ± 5.21	40.18 ± 4.10
	−20 min	39.50 ± 4.07	40.28 ± 5.13	41.87 ± 5.40
CMJ Height	+20 min	42.27 ± 5.12	41.85 ± 5.48	44.19 ± 4.63
	−20 min	42.33 ± 4.78	43.82 ± 4.77	44.71 ± 5.05
CMJ Free Height	+20 min	50.40 ± 6.68	52.81 ± 7.31	52.65 ± 6.13
	−20 min	51.43 ± 4.60	52.61 ± 5.74	52.82 ± 5.58

CMJ: countermovement jump.

## Data Availability

The data that support the findings of this study are available from the corresponding author, upon reasonable request.
